# Validation of the Swedish version of HLS_19_-Q12: a measurement for general health literacy

**DOI:** 10.1093/heapro/daaf132

**Published:** 2025-08-01

**Authors:** Josefin Wångdahl, Maria Jaensson, Karina Dahlberg, Lina Bergman, Roger Keller Celeste, Megan Doheny, Janne Agerholm

**Affiliations:** Aging Research Center, Department of Neurobiology, Care Sciences and Society, Tomtebodavägen 18a, 171 77 Stockholm, Sweden; Division of Nursing, Department of Neurobiology, Care Sciences and Society, Alfred Nobels Allé 23, C2, 141 52 Huddinge, Sweden; Department of Public Health and Care Sciences, Uppsala University, Box 564, 751 22 Uppsala, Sweden; Faculty of Medicine and Health, School of Health Science, Örebro University, Fakultetsgatan 1, SE-701 82 Örebro, Sweden; Faculty of Medicine and Health, School of Health Science, Örebro University, Fakultetsgatan 1, SE-701 82 Örebro, Sweden; Division of Nursing, Department of Neurobiology, Care Sciences and Society, Alfred Nobels Allé 23, C2, 141 52 Huddinge, Sweden; Aging Research Center, Department of Neurobiology, Care Sciences and Society, Tomtebodavägen 18a, 171 77 Stockholm, Sweden; Department of Preventive and Social Dentistry, Federal University of Rio Grande do Sul, Ramiro Barcelos 2492, 3rd Floor, 90035-003 Porto Alegre, Brazil; Aging Research Center, Department of Neurobiology, Care Sciences and Society, Tomtebodavägen 18a, 171 77 Stockholm, Sweden; Department of Global Public Health, Tomtebodavägen 18a, 171 77 Stockholm, Sweden; Aging Research Center, Department of Neurobiology, Care Sciences and Society, Tomtebodavägen 18a, 171 77 Stockholm, Sweden; Department of Global Public Health, Tomtebodavägen 18a, 171 77 Stockholm, Sweden

**Keywords:** health literacy, HLS19-Q12, M-POHL, measurements, validation, cognitive interviews, Sweden

## Abstract

Health literacy (HL) is a critical determinant of health, as it affects health behavior and outcomes, in addition to equity in health. This study aimed to translate, culturally adapt, and validate the Swedish version of HLS_19_-Q12 (HLS19-Q12-SE). The HLS_19_-Q12 is a widely used instrument that consists of 12-items and is often used to assess HL in Europe. A convergent mixed-methods design was applied, including cognitive interviews (*n*  *=*  *8*) and psychometric testing with a survey sample (*n*  *=*  *374*) and test–retest group (*n*  *=*  *28*). The instrument was forward and backwards translated and culturally adapted. Data was analyzed using confirmatory and exploratory factor analysis, correlation testing, and reliability assessments. Cognitive interviews explored the clarity, interpretation, and contextual relevance of the items. Participants found the HLS_19_-Q12-SE clear and easy to understand, though some reported difficulties with unfamiliar health situations and uncertainty of the term “health information.” Based on the interviews, the examples for one of the items were culturally adapted. Psychometric testing showed good feasibility, no floor or ceiling effects on scale level, and moderate positive correlations with other HL instruments, supporting internal and external validity. Structural validity was confirmed, and internal consistency and test–retest reliability were satisfactory. However, ceiling effects were observed at the dichotomous item level, and correlations with self-rated health and social status were low. The HLS_19_-Q12-SE is a reliable and valid instrument for assessing general HL in Sweden. While psychometric properties were strong overall, future research should address ceiling effects on item level and explore the instrument’s performance in more diverse populations.

Contribution to Health Promotion

- According to the World Health Organization, health literacy (HL) is one of the three pillars of health promotion.
- Measuring individual HL is essential for identifying groups at risk of health inequalities and to assess whether interventions benefit individuals with varying HL levels. Instruments also enable the evaluation of efforts aimed at improving HL. However, the usability of these measurements depends on how it is adapted to specific populations and contexts.
- Continuous validating of new versions of HL instruments is crucial to ensure accuracy, culturally relevance and effectiveness in different contexts.

## INTRODUCTION

Health literacy (HL) is a social determinant of health that significantly impacts empowerment, health outcomes, and equity in health (Nutbeam and Lloyd 2021). Over the past decade, there has been a paradigm shift in how the concept of HL is described. Previous definitions mainly focused on individuals’ ability to access and understand written health information (i.e. functional HL), but now the definition includes more skills, including those needed to use oral health information (i.e. general HL) (Sørensen 2019). In this study, we use the definition from Sørensen *et al*. (2012), which states that general HL is “linked to literacy and entails people's knowledge, motivation and competencies to access, understand, appraise, and apply health information to make judgments and take decisions in everyday life concerning healthcare, disease prevention, and health promotion to maintain or improve quality of life during the life course” (Sørensen *et al*. 2012).

In Europe, between 27% and 48% of the population were assessed to have limited HL (Baccolini *et al*. 2021), this is an issue because limited HL is associated with a range of adverse health outcomes, such as adopting unhealthier health behaviors (Sørensen *et al*. 2015, Svendsen *et al*. 2020, The HLS19 Consortium of the WHO Action Network M-POHL 2021), inappropriate or excessive use of healthcare services (Wångdahl *et al*. 2018, The HLS19 Consortium of the WHO Action Network M-POHL 2021) poorer self-management behavior (van der Gaag *et al*. 2022, Billany *et al*. 2023), lower participation in health promotion and disease prevention programs (Coughlin *et al*. 2020) and poorer health status (Sørensen *et al*. 2015, Wångdahl *et al* 2018, Svendsen *et al*. 2020, The HLS19 Consortium of the WHO Action Network M-POHL 2021). Given the negative effects of limited HL, it is essential to address HL to improve health outcomes (Pelikan *et al*. 2018). Measuring HL is vital because understanding HL levels across different subgroups within the population provides valuable insights into where targeted interventions are needed. From an equity perspective, it is essential to ensure that health interventions benefit all individuals in the target group, regardless of differences in HL. Additionally, accurate measurements of HL are needed to determine whether interventions aimed at promoting HL are producing the desired results.

During the last decade, several instruments have been developed (Tavousi *et al*. 2022). One is the HL Survey European Questionnaire, HLS-EU-Q47, a self-report instrument consisting of 47 questions measuring HL as defined by Sørensen *et al.* 2012. In 2013, this instrument was used in a study including populations from eight European countries (Sørensen *et al*. 2015). As a result of that study, the shorter 16-item version, HLS-EU-Q16, was developed (Sørensen *et al*. 2012). The HLS-EUQ47 and HLS-EU-Q16 have been translated into over 20 languages (Pelikan *et al*. 2022). Further, HLS-EU-Q16 has been translated into Swedish, tested, and is considered reliable and valid instrument (Bergman *et al*. 2023a, 2023b). However, in the development of the latest European HL Survey HLS_19_, researchers found that another even shorter form of the HLS_19_-Q12, better represented the underlying model of general HL. Since January 2024, the M-POHL network (WHO Action Network on Measuring Population and Organizational HL), the research consortium who conducts the European HL survey recommends the use of HLS_19_-Q12, and as such it has been translated, adapted, and psychometrically evaluated in several countries (Pelikan *et al*. 2022). However, to date, the HLS_19_-Q12 instrument has not yet been translated into Swedish or validated for use in Sweden. The aim of this study was to translate, culturally adapt and validate the HLS_19_-Q12 for a Swedish context.

## METHODS

### Study design

A validation study using data collected from cognitive interviews and psychometric tests to examine the HLS_19_-Q12 use for the Swedish context.

### Ethics

The study has been approved by the Swedish Ethical Review Authority, Sweden (no. 2023-02239-01), and follows the principles outlined in the 1964 Helsinki Declaration and its subsequent amendments. All participants received both oral and written information about the study and were given the opportunity to ask questions before participating. Informed consent was obtained through participation. The information material for both the cognitive interviews and the survey stated that participation, by taking part in the interview or completing and submitting the questionnaire, would be regarded as consent to participate in the study.

### Setting, sample, and data collection

Although there is no universally accepted rule for number of participants in psychometric validation studies, commonly cited guidelines suggest a minimum of 300 participants, or at least 10 individuals per item (Boateng *et al*. 2018). Given our 12-item instrument, we aimed at a sample size of at least 300 for this study. Participants had to be ≥ 18 years old, understand and complete the questions in Swedish, and present in-person the day of data collection. Convenience sampling was used to recruit participants from different arenas, where people with different characteristics in terms of gender, age, and educational level could be reached. Specifically, data were gathered from soldiers, factory workers, healthcare and nursing staff, individuals working within the Swedish church, choir singers, amateur athletes, and active retirees across 15 different workplaces and nongovernmental organizations. Detailed notes were taken throughout the data collection to identify the types of individuals from whom data was collected in each arena. Based on this, subsequent arenas were selected to ensure a varied and representative sample of participants.

Each arena was contacted, and if consent for data collection was given, groups with potential participants were informed about the study and asked to participate. Depending on the arena, the information was first distributed by email and then verbally by the contact person from the arena or verbally and in writing by the researcher in the project. At least one day’s reflection time regarding participation in the study was given before data collection was carried out. On the day of data collection, one of the researchers or the contact person at the arena distributed and collected the questionnaire onsite, with except for one arena. There, participants were given an empty envelope together with the questionnaire, which they used to submit their completed questionnaire a few days later. This special procedure was implemented to allow participants to consider whether to participate, even though information about the study and questionnaire had been provided at the same time at this arena. Most of those invited to participate in the study were interested and wanted to take part. Of those who declined, most did not have the time or did not feel they were suited for the study.

Most participants in the study answered the study’s questionnaire once (the test group, *n* = 374). However, a subgroup of the participants (the test–retest group, *n* = 28) answered the study’s questionnaire twice (1 week apart). The latter’s size was based on the recommendation that the appropriate size for a test–retest group in reliability testing is at least 25 people (Bujang and Baharum 2017). To avoid the collection of personal data but at the same time to be able to match data from the two measurement occasions in the test–retest group at the individual level, the participants marked their questionnaires with a code consisting of the first three letters of their mother’s name and her year of birth. Data collection were carried out from June to October 2023. In addition, eight additional people were recruited to participate in cognitive interviews on the Swedish version of the HLS_19_-Q12 instrument. Convenience sampling and the researchers’ networks were used to find accessible participants to these interviews with different socio-demographic characteristics—equal numbers of women and men with Swedish as their native language participated.

### Questionnaire

For external validation, the questionnaire included three HL instruments: the HLS_19_-Q12, the Swedish version of the Functional Health Literacy scale (S-FHL), and the Swedish version of the Communicative and Critical Health Literacy scale (S-CCHL), along with questions on self-rated health, internet use, and socio-demographic characteristics. The S-FHL and S-CCHL were included because they are among the few HL instruments that have been translated into Swedish and demonstrated good validity in the Swedish context (Wångdahl and Mårtensson 2014, Mårtensson and Wångdahl 2017, Jaensson *et al*. 2021). Additional reasons for their inclusion were that they assess key HL skills encompassed by the broader construct of general HL, consist of a relatively small number of items, and are commonly used in Swedish HL research.

*The HLS_19_-Q12* (Table 2) capture four aspects of managing health information (access, understand, appraise, and apply) within three domains (health promotion, disease prevention, and health care) (Pelikan *et al*. 2022) and was developed within “HLS_19_—the International HL Population Survey 2019-2021” of M-POHL (m-pohl.net/HLS19). It consists of 12 items, which all are answered by a 4-point ordinal Likert scale ranging from 1 (Very difficult) to 4 (Very easy). The HLS_19_-Q12 index score was based on polytomous items and obtained by calculating the mean of the values of the items filled in scaled from 0 to 100. At least 80% of the items should be answered; if not, the calculated score is treated as missing. Depending on the calculated HL index score, a person is assessed as having excellent (>83.33), sufficient (>66.67 and ≤83.33), problematic (>50 and ≤66.67), or inadequate HL (≤50) (Pelikan *et al*. 2022). The index can also be based on dichotomized items where the values 1 and 2 are coded as zero, and values 3 and 4 are coded as one. The scale is also rated from 0 to 100 (number of responses coded as 1/the number of valid responses * 100). The scoring based on dichotomized items was in this study only used for supplementary analyses in the explorative factor analyses. The HLS_19_-Q12 can be used by third parties for research purposes free of charge but requires a contractual agreement between the user and the ICC of the HLS_19_ Consortium, for more information see: https://m-pohl.net/HLS19Instruments.

“The S-FHL scale” assesses functional HL, that is, an individual’s ability to understand and use written health information (Mårtensson and Wångdahl 2017) and was originally developed in Japan (Ishikawa Takeuchi and Yano 2008). It consists of five items: two focusing on visual ability, two on understanding words and concepts, and one on needing help from others to access health information. All are answered by a 5-point ordinal scale ranging from 1 (never) to 5 (always), with higher scores indicating lower HL. According to the scale guidelines, the S-FHL scores were calculated and classified as inadequate, problematic, or sufficient HL (Mårtensson and Wångdahl 2017).

“The S-CCHL scale” assesses communicative and critical HL, that is, an individual’s ability to communicate, critically analyze and use various health information (Wångdahl and Mårtensson 2014) and was originally developed in Japan (Ishikawa *et al*. 2008) It consists of five items: three focusing on the ability to collect, extract, and understand health information and two on assessing and applying health information. All are answered by a 5-point ordinal scale ranging from 1 (“strongly disagree”) to 5 (“strongly agree”), with higher scores indicating lower HL. According to the scale guidelines, the S-CCHL scores were calculated and classified as inadequate, problematic, or sufficient HL (Wångdahl and Mårtensson 2014).

“Self-rated health” was assessed by the question “How do you assess your overall health status?” with five response options ranging from “very poor” to “very good” (McDowell 2010). This question is one of the most frequently used measures of self-rated health and has shown to have satisfactory validity in many countries (Baćak and Ólafsdóttir 2017). The Swedish version has been demonstrated to have good validity (Lundberg and Manderbacka 1996) and is used in the national public health survey (The Public Health Agency of Sweden 2023).

“Socio-demographic questions” were asked about “age, biological sex, highest level of education, and socioeconomic position.” However, since it was not possible to differentiate between individuals at higher levels of education (56% was categorized as having a high educational level), we in the end decided not to use this variable in the analyses. “Socioeconomic position” was based upon occupation and categorized into three levels according to the Swedish version of the International Standard Classification of Occupations also called SSYK (Swedish acronym): Low (Occupations demanding education up to the level of post-secondary education shorter than 2 years), Middle (occupations demanding practical or profession-specific post-secondary education in 2–3 years) and High (Management professions and occupations demanding theoretical or research preparatory post-secondary education and postgraduate education of at least 3 years, normally 4 years or longer) (Statistics Sweden 2012). This question is regularly used in Swedish national statistics and survey research.

### Translation

The English version of HLS_19_-Q12 was translated to Swedish in line with M-POHL’s guidelines for translation and cultural adaptation of the instrument, which included several steps (The HLS19 Consortium of the WHO Action Network M-POHL 2021). (i) Two forward translations were performed, one by the research group and one by a professional translator. In the research group, the first author, is fluent in English, has vast experience in translations of HL instruments into Swedish, and carried out the translation. Both she and the professional translator are native Swedish speakers. (ii) The research team compared the translations, and the most appropriate translations were selected for each item. The translations were chosen based on criteria ensuring the language was easy to understand, not overly academic, and grammatically correct. Some items used the research team’s translations, others used the professional translator’s, and some combined both. (iii) The preliminary Swedish version was shared with a “Swedish for Beginners” teacher who reviewed the translation, commented on the extent to which plain language was used, and made suggestions for improvement. (iv) A Norwegian researcher, knowledgeable in Swedish, who has worked on the translation of the Norwegian HLS_19_-Q12 investigated how the Swedish preliminary version matched the Norwegian version and to what extent the content of the questions was in line with what they aim to measure according to the M-POHL consortium. (v) A Danish Swedish-speaking researcher in our team compared the Swedish preliminary version with the Danish HLS_19_-Q12 version. (vi) Some minor linguistic adjustments were made regarding vocabulary and grammatical structure based on wording in the Norwegian and Danish versions. (vii) The final Swedish version was back-translated into English by the first author using the online translation program DeepL. Thereafter, the first and last author compared back-translation with the original English version of the HLS_19_-Q12. And together, judged that the translation was sufficiently similar to the original.

### Cognitive interviews

All interviews were conducted in a private undisturbed place and at a time chosen by the selected respondents. The meeting began with verbal information about the study and with the informant giving informed consent to participate. The respondent was then given the Swedish version of the HLS_19_-Q12 (HLS_19_-Q12-SE) in paper format and instructed to answer the items while explaining aloud how they interpret the items and answer options and what they thought when reading the initial instructions and answering the items. While the respondent was doing this, the interviewing researcher carefully wrote down what the respondent said. When the participants had finished, they were asked to clarify their thoughts about some of the questions in cases where they had given less detail about how they had interpreted or reasoned about an item and its answer. The interviews lasted between 20 and 35 min and were not recorded.

### Psychometric testing and data analysis

The selection of psychometric tests was guided by the COnsensus-based Standards for the selection of health Measurement INstruments (COSMIN) (Mokkink *et al*. 2016). The number and percentages of data, along with their mean, standard deviation (SD), or range are presented, depending on the data type. Two-tailed *P*-values <0.05 were statistically significant. IBM SPSS Statistics version 28.0 and Mplus 7.1 was used for the psychometrics tests.

#### Feasibility, floor, and ceiling effects

Feasibility was assessed by examining the proportion of participants with a valid HLS_19_-Q12 index score and the response rate for each item. A response rate of at least 95% on the index scale and individual items was considered satisfactory (Mokkink *et al*. 2019). Floor and ceiling effects were examined on HLS_19_-Q12 index score and at the item level. If <15% of participants had the lowest or highest possible value on the index scale, respectively, on the individual questions, the floor and ceiling effect was considered satisfactory (De Vet *et al*. 2014).

#### Internal construct validity

Confirmatory factor analysis (CFA) and exploratory factor analysis (EFA) were conducted to assess the structural validity of the scale. CFA was used to test the fit of the original one-factor model and to compare the performance of ordinal and dichotomous items, and EFA was used to examine the dimensionality of the scale, CFA was performed using the weighted least squares means and variance adjusted estimation method. Model fit was evaluated using standard indices: comparative fit index (CFI), Tucker–Lewis index (TLI), root mean square error of approximation (RMSEA), and weighted root mean square residual (WRMR). The following thresholds were used to assess model fit: CFI and TLI ≥ 0.95 (good fit), RMSEA ≤ 0.05 (good fit), and WRMR ≤ 1.00 (Byrne 2012). WRMR values below or close to 1 indicate good fit, although WRMR may increase with model misspecification and sample size.

Standardized factor loadings ranging from 0.35 to 0.50 are considered fair, and ≥0.50 were considered strong loading to support (Reichenheim *et al*. 2014). To further assess item performance and the hierarchical ordering of the HLS_19_-Q12, a scalability analysis was conducted based on the proportion of item endorsement across total HL scores. Finally, internal consistency was assessed with Cronbach’s alpha and McDonald’s omega. McDonald’s omega was included, as it provides a robust estimate of internal consistency when the assumption of tau-equivalence is violated. The EFA was performed using Horn’s Parallel Analysis with 500 iterations, based on polychoric correlations for ordinal items and tetrachoric correlations for dichotomized items. An iterated principal factors estimator was applied.

#### External construct validity

External validity was further assessed using Spearman’s rank coefficient (Mokkink *et al*. 2010) examining how the HLS_19_-Q12 index correlated with other HL instruments and variables. We hypothesized that the HLS_19_-Q12 index score would correlate with both S-FHL and S-CCHL because functional, communicative, and critical HL are included in general HL. Furthermore, we hypothesized that the HLS_19_-Q12 index score would positively correlate with good self-perceived health and high socioeconomic position since those associations have been found in research previously (Sørensen *et al*. 2015, Svendsen *et al*. 2020, The HLS19 Consortium of the WHO Action Network M-POHL 2021). The following thresholds were used to assess how satisfactory the magnitude of the correlation coefficient was: 0 < 0.1 (negligible), 0.1–0.39 (weak), 0.4–0.69 (moderate), 0.7–0.89 (strong), and 0.9–1.0 (very strong) (Schober *et al*. 2018).

#### Reliability

Split-half reliability was evaluated using the Spearman–Brown coefficient. Internal consistency values above 0.70 were considered acceptable for group-level research use (Terwee *et al*. 2007). Test–retest reliability was evaluated by calculating the quadratic weighted Cohen’s Kappa coefficient between the HLS_19_-Q12 index scores at two time points (*n* = 26). A value >0.70 was considered satisfactory (Terwee *et al*. 2007).

## RESULTS

### Cognitive interviews

Eight cognitive interviews were conducted. Participants included five women and three men; half had a university degree, and the others had primary or secondary education. Five were aged 20–50 and three were 70–80, to reflect perspectives across different age groups. Most participants reported that the introduction with instructions about the instrument and how to answer the questions was clear. The instrument’s language was perceived as simple, well-formulated, and easy to understand. However, some felt that they had to reread the introduction multiple times, as the first part of each question referred back to it. At the same time, most participants noted that it was unclear what type of health information was being referred to, and that their answer depended on both the type of information and its source. In addition, participants reported that it was difficult to answer questions about situations they had not experienced. Most commonly, the participant chose the answer option “difficult” or refrained from answering these items altogether. This problem mainly concerned three items dealing with health information related to illness and/or treatment: c. … to assess the advantages and disadvantages of different treatment options; f. … to understand information about recommended health examinations? (e.g. screening for colorectal cancer, measuring blood sugar, or blood pressure); and h. … determine how to protect themselves from diseases using information from the mass media? (e.g. from newspapers, TV, or the Internet). Another finding was that participants who reasoned similarly about an item, and therefore, should have chosen the same response options, ended up selecting different ones. Based on the interviews, no major changes were made to the items. However, the examples in item “f” were changed to “e.g. mammography, screening for colon, and rectal cancer.”

### Psychometric properties

#### Demographics of the psychometric test sample

A total of 374 respondents participated. The estimated response rate varied between 60% and 80% in the different settings, with participants from working places having a lower response rate than participants from leisure associations. The majority (67%) were female. The mean age for the total sample was 51.7 years (range 18–91 years); 31% were categorized as high, 28% as middle, and 41% as low socioeconomic position. The majority perceived their health to be good (*n* = 169; 46%) or very good (*n* = 108; 29%). The mean score for HLS_19_-Q12-SE was 72.6 (range 19.4–100) and there was an almost equal distribution of participants classified as having inadequate (7.4%), problematic (36.3%), sufficient (29.0%), and excellent (27.3%) HL (see Table 1).

**Table 1.** Demographics of the respondents with valid responses and the test–retest group.

	Total sample (*n* = 374)	Test–retest group (*n* = 28)
Gender, *n* (%)		
Male	122 (32.6)	9 (32.1)
Female	251 (67.1)	19 (67.9)
Age in years		
Mean (SD)	51.7 (21.0)	56.1 (17.3)
Range	18–91	(29–84)
Socioeconomic position, *n* (%)		
Low	142 (41.2)	1 (3.7)
Middle	97 (28.1)	11 (40.7)
High	106 (30.7)	16 (59.3)
General self-perceived health, *n* (%)		
Very poor	6 (1.6)	4 (14.3)
Poor	25 (6.8)	10 (35.7)
Fair	61 (16.5)	4 (14.3)
	169 (45.8)	5 (17.9)
Very good	108 (29.3)	5 (17.9)
S-FHL,^a^ *n* (%)		
Inadequate	62 (17.3)	3 (11.5)
Problematic	164 (45.7)	10 (38.5)
Sufficient	133 (37.0)	13 (50.0)
S-CCHL,^b^ *n* (%)		
Inadequate	41 (11.4)	1 (3.6)
Problematic	153 (42.4)	8 (28.6)
Sufficient	167 (46.3)	19 (67.9)
HLS_19_-Q12-SE^c^ levels, *n* (%)		
Inadequate	27 (7.4)	2 (7.1)
Problematic	133 (36.3)	9 (32.1)
Sufficient	106 (29.0)	10 (35.7)
Excellent	100 (27.3)	7 (25.0)
HLS_19_-Q12-SE^c^ score		
Mean (SD)	72.62 (16.1)	73.9 (17.3)
Range	19.44–100	27.78–100

^a^The Swedish Functional Health Literacy Scale.

^b^The Swedish Communicative and Critical Health Literacy Scale.

^c^The Swedish Health Literacy Survey European Questionnaire 12 items.

For the test–retest group (*n* = 28), most of the respondents were female (68%) and the mean age for the total sample was 56.1 years (range 29–84). In this group, the distribution of socioeconomic position was 59% in high, 41% in middle, and only 4% (*n* = 1) in low socioeconomic position. The mean score for HLS_19_-Q12-SE was 73.9 (range 27.8–100), and also in this group, there was equal distribution of participants classified as having problematic (32%), sufficient (36%), and excellent (25%) HL (Table 1).

#### Feasibility and floor and ceiling effects

No floor effects were detected on item level in the ordinal version, but 11 of 12 items showed ceiling effects (i.e. >15% of respondents scored the highest). All items except two (items 4 and 10) had complete variance (i.e. at least one respondent for each scoring option. All items had <5% missing. Table 2 presents item distributional statistics for each item of HLS_19_-Q12-SE. At the scale level, the response rate was 98% and no floor or ceiling effects were identified (not presented in any table). No respondent had the lowest HLS_19_-Q12-SE index score and six respondents (5%) had the highest index score.

**Table 2.** Descriptive statistics for division of valid respondents on HLS_19_-Q12-SE.

HLS_19_-Q12 items^a^	All (*n* = 374)
	*n* (%)
1. Find out where to get professional help when you are ill. (*n* = 371)	
Very difficult	3 (0.8)
Difficult	34 (9.2)
Easy	168 (45.3)
Very easy	166 (44.7)
2. Understand information about what to do in a medical emergency. (*n* = 369)	
Very difficult	3 (0.8)
Difficult	31 (8.4)
Easy	187 (50.7)
Very easy	148 (40.1)
3. Judge the advantages and disadvantages of different treatment options. (*n* = 367)	
Very difficult	10 (2.7)
Difficult	155 (42.3)
Easy	151 (41.1)
Very easy	51 (13.9)
4. Act on advice from your doctor or pharmacist. (*n* = 369)	
Very difficult	0 (0)
Difficult	23 (6.2)
Easy	190 (51.5)
Very easy	156 (42.3)
5. Find information on how to handle mental health problems (*n* = 363).	
Very difficult	15 (4.1)
Difficult	95 (26.2)
Easy	165 (45.5)
Very easy	88 (24.2)
6. Understand information about health screenings and examinations (*n* = 360).	
Very difficult	10 (2.8)
Difficult	77 (21.4)
Easy	171 (47.5)
Very easy	102 (28.3)
7. Judge if information on unhealthy habits, such as smoking low physical activity or drinking too much alcohol, are reliable (*n* = 370).	
Very difficult	4 (1.1)
Difficult	37 (10.0)
Easy	163 (44.1)
Very easy	166 (44.8)
8. Decide how you can protect yourself from illness using information from the mass media (*n* = 368).	
Very difficult	13 (3.5)
Difficult	81 (22.0)
Easy	168 (45.7)
Very easy	106 (28.8)
9. Find information on healthy lifestyles such as physical exercise, healthy food or nutrition (*n* = 373).	
Very difficult	2 (0.5)
Difficult	13 (3.5)
Easy	156 (41.8)
Very easy	202 (54.2)
10. Understand advice concerning your health from family and friends (*n* = 364).	
Very difficult	0 (0)
Difficult	27 (7.4)
Easy	178 (48.9)
Very easy	159 (43.7)
11. Judge how your housing conditions may affect your health and well-being (*n* = 369).	
Very difficult	5 (1.4)
Difficult	52 (14.1)
Easy	175 (47.4)
Very easy	137 (37.1)
12. Make decisions to improve your health and well-being (*n* = 371).	
Very difficult	4 (1.1)
Difficult	69 (18.6)
Easy	161 (43.4)
Very easy	137 (36.9)

The HLS_19_-Q12 can be used by third parties for research purposes free of charge but requires a contractual agreement between the user and the ICC of the HLS_19_ Consortium, for me information see: https://m-pohl.net/HLS19Instruments.

^a^Ingress for the HLS_19_-Q12-SE: “It is not always easy to get understandable, reliable, and useful information on health-related topics. With the following questions we would like to find out which tasks related to handling health information are more or less easy or difficult. On a scale from very easy to very difficult, how easy would you say it is to…”

#### Internal construct validity

Table 3 shows the results from the CFA. For the ordinal model, fit indices were: RMSEA = 0.10, CFI = 0.96, TLI = 0.95, and WRMR = 1.27. The fit of the dichotomous model was slightly better with RMSEA = 0.04, CFI = 0.96, TLI = 0.95, and WRMR = 0.95. The χ^2^ tests were statistically significant in both models; however, this was expected given that large sample. Standardized factor loadings in the dichotomous model ranged from 0.56 to 0.82, all above the acceptable threshold. Internal consistency was satisfactory in both models, with McDonald’s omega = 0.79 (dichotomous) and 0.90 (ordinal), and Cronbach’s alpha = 0.79 (dichotomous) and 0.90 (ordinal), indicating acceptable to good reliability.

**Table 3.** Factor loadings of HLS_19_-Q12-SE in CFA with ordinal and dichotomous items.

	Initial 1-factor model (Dichotomous items)		Initial 1-factor model (Ordinal items)	
F1 BY	STDYX Coeff	Uniqueness	STDYX Coeff	Uniqueness
Item 1	0.69	0.52	0.67	0.55
Item 2	0.82	0.33	0.74	0.45
Item 3	0.79	0.38	0.78	0.39
Item 4	0.77	0.40	0.74	0.45
Item 5	0.72	0.48	0.73	0.47
Item 6	0.75	0.44	0.76	0.43
Item 7	0.69	0.52	0.73	0.48
Item 8	0.68	0.53	0.69	0.53
Item 9	0.73	0.46	0.76	0.42
Item 10	0.70	0.52	0.75	0.44
Item 11	0.56	0.69	0.68	0.54
Item 12	0.62	0.62	0.69	0.53
Fit Indices:				
df	54	–	46	–
x2	93.803	–	249.405	–
*P*-value	<0.001	–	<0.001	–
RMSEA	0.04	–	0.10	–
WRMR	0.95	–	1.27	–
CFI	0.96	–	0.96	–
TLI	0.95	–	0.95	–
McDonald’s omega	0.79	–	0.90	–
Cronbach’s alpha	0.79	–	0.90	–

EFA was conducted as a complementary assessment of the dimensionality of HLS_19_-Q12-SE. For the ordinal model, the adjusted eigenvalue for the first factor was 6.39, compared with 0.85 for the second factor. This gives a ratio of 7:1. Similarly, in the dichotomous model, the adjusted eigenvalues were 6.03 for the first factor and 1.24 for the second giving a ratio close to 5:1 (see Supplementary Table S1). This exceeds the rule-of-thumb that the first eigenvalue should be at least four times the second to indicate unidimensionality and supports the assumption that the HLS_19_-Q12-SE measures a single underlying construct of general HL (Reichenheim and Bastos 2021). A scalability analysis was conducted to examine how item endorsement levels varied across total HL scores, providing further insight into the scale’s hierarchical structure. Items were ordered from easiest to most difficult based on their overall proportion of positive responses, which ranged from 96% (Item 9) to 55% (Item 3) (see Supplementary Table S2). In general, the proportion of positive responses increased with higher total HL scores, supporting the expected monotonic pattern for a unidimensional scale.

#### External construct validity

No significant correlations were observed between HLS_19_-Q12-SE and social status, and self-perceived general health. A moderate positive correlation was detected between HLS_19_-Q12-SE, S-FHL, and S-CCHL (Table 4).

**Table 4.** Spearman rho correlations between HLS_19_-Q12-SE, socioeconomic position, self-perceived health, S-FHL, and S-CCHL.

Variables	1.	2.	3.	4.	5.
1. HLS_19_-Q12-SE score^a^	1.000	–	–	–	–
2. Socioeconomic position^b^	0.062	1.000	–	–	–
3. Self-perceived general health^c^	0.115*	0.065	1.000	–	–
4. S-FHL^d^	0.511**	0.090*	0.155**	1.000	–
5. S-CCHL^e^	0.555**	0.246**	0.059	0.410**	1.000

^a^HLS_19_-Q12-SE index score, range 0–100.

^b^Socioeconomic position, 1 = low, 2 = middle, and 3 = high.

^c^Self-perceived general health, 1 = very poor, 2 = poor, 3 = fair, 4 = good, and 5 = very good.

^d^S-FHL, 1 = inadequate, 2 = problematic, and 3 = sufficient.

^e^S-CCHL, 1 = inadequate, 2 = problematic, and 3 = sufficient. *Correlation is significant at the 0.05 level (2-tailed). **Correlation is significant at the 0.01 level (2-tailed).

#### Reliability

HLS19-Q12-SE demonstrated acceptable internal consistency, with a Spearman–Brown coefficient ≥0.7 (0.82). Test–retest reliability of the HLS19-Q12-SE score was also acceptable (≥0.7), with a Cohen’s Kappa of 0.7. Consistency over time between HLS19-Q12-SE levels was supported by a Cohen’s Kappa of 0.66 (Table 5).

**Table 5.** Reliability statistics for HLS_19_-Q12-SE (spearman brown-coefficient, *n* = 335; Cohen κ, *n* = 28).

Variables	Spearman Brown-coefficient	Cohen’s κ
HLS_19_-Q12-SE Score	0.822	0.715^a^
HLS_19_-Q12-SE Levels	–	0.656^a^

^a^Significance at the 0.001 level (quadratic).

## DISCUSSION

The aim of the present study was to translate, culturally adapt, and evaluate the validity of the HLS_19_-Q12 for a Swedish context.

### Cognitive interviews

The cognitive interviews indicated that the HLS_19_-Q12-SE was generally perceived as clear and easy to understand. However, some participants reported difficulty answering questions related to health situations they had not personally experienced. A similar issue was observed in a previous validation study of the HLS-EU-Q47, the longer version of the same instrument conducted among adolescents (Domanska *et al*. 2018). This suggests that respondents may need to estimate how easy or difficult a task would be based on their experiences in other areas of health, which could affect the validity of the instrument. Nevertheless, the psychometric evaluation was satisfactory. Another issue was that some participants were uncertain about how to interpret the term “health information”—specifically, what types of information were included and at what level of complexity. For instance, some were unsure whether the questions referred to general, easy-to-understand information or more complex medical content. This variation in interpretation suggests that respondents may base their answers on different understandings of what “health information” entails. This could potentially influence their choice of response and therefore the final HL index score and the comparability of HL across individuals. Despite the reported variations, we chose not to revise the wording on this occasion. Providing a more detailed definition might unintentionally narrow respondents’ interpretations and could restrict the instrument’s applicability for people with different experiences of health information. Also, altering the wording would reduce comparability with the original version of the instrument. On the other hand, we did identify a need to adapt the examples in item f to better suit the Swedish context. We suggest that the examples “e.g. screening for colorectal cancer, measuring blood sugar or blood pressure” are replaced with “e.g. mammography, screening for colon and rectal cancer” when using HLS_19_-Q12-SE in the future. This recommendation is because blood tests and blood pressure measurements are typically part of routine check-ups rather than organized screening programs in Sweden. Hence, they do not usually require individuals to actively decide whether to participate—this distinction was also highlighted by participants during the cognitive interviews. We have only been able to identify one validation study of the HLS_19_-Q12, which also included results from cognitive interviews. In the translation and development of the Chinese version of HLS_19_-Q12 several minor cultural changes were made after the initial pilot testing of the instrument, but also primarily in the examples given for each item. In 8 of 12 items, examples after the statements were added or clarified (Liu *et al*. 2024). In their version, they added blood pressure test, where in our version we have excluded it, as this is not part of any screening program in Sweden. The relatively few adaptations required in the Swedish version may be due to the cultural adjustments made during the translation phase, where we modified words and examples to better fit the Swedish context. Cognitive interviews have also been made for other short versions of the HLS-EU-Q47 instrument. Both a different Q12 version (Muñoz-Villaverde *et al*. 2024), where six questions overlap in the two Q12 versions, and a Q16 version (Gustafsdottir *et al*. 2020) where six items overlapped with the Q12 version from this study as well. From these validation studies, only a few changes were made after the cognitive interviews, but not in the item that we had to adjust.

### Psychometric properties

Our findings from the psychometric testing show that the HLS_19_-Q12-SE demonstrated generally strong psychometric properties supporting unidimensionality of the scale, comparable with the results from the validation statistics of the instrument in 17 other European Countries (Pelikan *et al*. 2022). At the scale level, the HLS_19_-Q12-SE demonstrated good feasibility, with low levels of missing data and no floor or ceiling effects. While previous studies have reported ceiling effects when using dichotomized scoring methods (Pelikan *et al*. 2022), the studies we identified that used polytomous scoring found no floor or ceiling effects at the scale level (Pelikan *et al*. 2022, Touzani *et al*. 2024). Ceiling effects were observed on items level, potentially limiting the instrument’s ability to differentiate among individuals with higher HL levels. While responses were still distributed across all categories, the absence of responses at certain score levels (e.g. scores 1 and 3) restricted scalability analysis and hindered a full assessment of item ordering across the range of scores. As previous studies have not reported on item-level ceiling effects, it is unclear whether this pattern is specific to the Swedish context or a feature of the instrument; however, item-level ceiling effects have also been reported in a Swedish study using the HLS-EU-Q16 (Bergman *et al*. 2023a, 2023b). A larger sample is needed in future studies in order to perform an IRT assessment where individuals score in all possible values. External construct validity was partially supported, with moderate correlations observed between the HLS_19_-Q12-SE and related HL instruments (S-FHL and S-CC HL). In the Chinese validation study, the instrument showed acceptable correlations when tested against the HLS-9 (Liu *et al*. 2024). Similarly, in the Portuguese version of the HLS_19_-Q12, associations were reported with both digital and navigational HL (Arriaga *et al*. 2022). In a European validation study conducted across 17 countries, the HLS_19_-Q12 demonstrated a strong correlation with the HLS_19_-Q47 (*r* = 0.897) (Pelikan *et al*. 2022), supporting its validity across different contexts. There were minimal correlations observed between self-perceived health and social status. This may be explained by several factors. First, the sample included a relatively high proportion of participants with good or very good self-rated health, which may have limited the variation needed to detect significant associations. Second, previous research suggests associations between HL, adverse health outcomes, and social gradients. These relationships are often complex and mediated by additional factors such as social and economic position, social networks, and health behaviors (Nutbeam and Lloyd 2021). Moreover, the purpose of this study was not to assess HL levels *per se*, but to evaluate the performance of the HLS19-Q12-SE in relation to established instruments. The observed weak correlations with health and socioeconomic position do not necessarily indicate poor construct validity. Future studies involving more diverse populations, including individuals with poorer health status and lower socioeconomic positions, are needed to explore these associations further. The results from both CFA and EFA support the structural validity. Model fit was acceptable to good for both the ordinal and dichotomous versions, with a slightly better fit for the dichotomous model. The EFA further confirmed the unidimensionality of the scale, indicating that HLS_19_-Q12-SE measures a single underlying construct of general HL. This is in line with other international validations of the instrument that supports a unidimensional structure of the instrument (Arriga *et al*. 2022, Pelikan *et al*. 2022, Liu *et al*. 2024). Cronbach’s alpha values in our study were similar to those reported in previously HLS_19_-Q12 studies: Chinese version 0.93 (Liu *et al*. 2024), Portuguese version 0.90 (Arriga *et al*. 2022) and between 0.80 and 0.90 (polytomous items) in the study by Pelikan *et al*. 2022 including 17 countries in Europe (Pelikan *et al*. 2022). In our study, test–retest reliability was satisfactory, with acceptable agreement over time, supporting the instrument’s stability. Evidence of stability over time for HLS19-Q12 is limited. We were only able to identify one other study examining stability; the Japanese version, which demonstrated high-level of test–retest reliability, however, this used a different version of the HLS_19_-Q12 where only half of the items overlap with the items in this version (Matsuo *et al*. 2024).

### Strengths and limitations

The strength of this study is the uses of comprehensive psychometric testing guided by the COSMIN framework (Mokkink *et al*. 2016) covering structural and construct validity, internal consistency, and test–retest reliability. The use of both exploratory and CFA provided robust evidence for the structural validity and unidimensionality of the HLS_19_-Q12-SE. In addition, the use of a relatively large and diverse sample enhanced the statistical power and stability of the findings. Another strength of this study was the rigorous translation and cultural adaptation process, which followed M-POHL guidelines and included two forward translations, cross-cultural comparisons with other Nordic versions, and feedback from plain-language experts. This process supported the cultural relevance of the Swedish version, while keeping the intended meaning of the items from the original instrument. Further, face validity was assessed through cognitive interviews with participants with various age and educational backgrounds. Through these interviews we identified interpretive challenges and got suggestions for. Another methodological strength is the use of polytomous scoring rather than dichotomized responses. This approach allowed for more nuanced response patterns and likely enhanced the sensitivity of the instrument, particularly among individuals with lower levels of HL. We also compared formally the psychometric properties of both versions, ordinal and dichotomous. However, several limitations must also be acknowledged. Although participants were recruited from diverse settings to capture s socio-demographic variation, the use of convenience sampling may limit the external generalizability. Moreover, there study included a relatively low proportion of male participants. An additional limitation of convenience sampling is not being able to determine the total number of people that contact persons asked to participate or the proportion who declined. Further, given that many participants were in good health and rated their HL as problematic or sufficient, we suspect that those with poorer health and very low HL may have declined. This, combined with the recruitment procedure, may be contributing to selection bias in this study. Most individuals who participated in the cognitive interview were native Swedes from affluent areas, which may further limit the generalizability of the findings to the overall population of Sweden. Typically, individuals from affluent areas, are well-educated and long-time residents, and consequently, are likely to have a better understanding of the Swedish healthcare system, and how to access services. They are also more knowledgeable about health promotion and disease prevention measures, which are widely available and affordable in Sweden. On the other hand, limited HL is more common among migrants (Baumeister *et al*. 2023). Additionally, cultural differences can shape how health is defined and perceived (Napier *et al*. 2014). It is widely acknowledged that lower socioeconomic and social status is often associated with having lower HL (Nutbeam and Lloyd 2021). Consequently, interpretation and responses to the questions may differ among individuals from lower socioeconomic and migrant-dense areas, compared with the study sample.

## CONCLUSION

HLS_19_-Q12-SE showed good psychometric characteristics and appears to be a reliable and valid instrument for assessing general HL in the adult Swedish population. Structural validity was supported through multiple analytic approaches, and both internal consistency and test–retest reliability were satisfactory. The scale also showed good feasibility, with low levels of missing data. However, ceiling effects were observed at the item level, and some expected correlations were not confirmed. Further research is needed to explore its responsiveness and applicability in more diverse populations, particularly among individuals with lower self-rated health and lower educational background.

## Supplementary Material

daaf132_Supplementary_Data

## Data Availability

The data underlying this article will be shared on reasonable request to the corresponding author.
