# Supplementary material for: Validation of the Swedish version of HLS19-Q12: a measurement for general health literacy
Source: Health Promot Int. 2025 Aug 1;40(4):daaf132. doi: 10.1093/heapro/daaf132 (PMC12314267; doi:10.1093/heapro/daaf132)
Supplement: daaf132_Supplementary_Data [file daaf132_supplementary_data.zip › Supplementary table 1b_Q12 validation study_250512.docx]

|  |  |  |  |
| --- | --- | --- | --- |
| **Supplementary table 1b:** Exploratory Factor Analysis based on tetrachoric correlations, and iterated principal factors estimator (dichotomous items) | | | |
| Estimation of Eigenvalues based on Horn's Parallel Analysis with 500 iterations | | | |
| Component | Adjusted | Unadjusted | Estimated |
| or Factor | Eigenvalue | Eigenvalue | Bias |
| 1 | 6.03 | 6.39 | 0.36 |
| 2 | 1.24 | 1.42 | 0.18 |
| OBS: if the first eigenvalue is >4 times the value of the second eigenvalue, there is very strong evidence of unidimensionality, independently of the value of the second eigenvalue | | | |
