# Supplementary material for: Validation of the Swedish version of HLS19-Q12: a measurement for general health literacy
Source: Health Promot Int. 2025 Aug 1;40(4):daaf132. doi: 10.1093/heapro/daaf132 (PMC12314267; doi:10.1093/heapro/daaf132)
Supplement: daaf132_Supplementary_Data [file daaf132_supplementary_data.zip › Supplementary table 2_Q12 validation study_250512.docx]

**Supplementary table 2.** Probability of item scoring according to HLS_19_-Q12-SE index score

|  |  |  | Endorsement by item (do you find it easy?) | | Proportion of positive items by HLS_19_-Q12-SE index score | | | | | | | | | | | | |
| --- | --- | --- | --- | --- | --- | --- | --- | --- | --- | --- | --- | --- | --- | --- | --- | --- | --- |
|  | Item | n | cases | % | 0 | 1 | 2 | 3 | 4 | 5 | 6 | 7 | 8 | 9 | 10 | 11 | 12 |
| Easiest | Item 9 | 373 | 358 | 96% | 0.0 | 0.0 | 25 | 0 | 40 | 63 | 82 | 83 | 93 | 95 | 91 | 100 | 100 |
|  | Item 4 | 369 | 346 | 94% | 0.0 | 0.0 | 25 | 0 | 40 | 57 | 82 | 85 | 100 | 93 | 98 | 100 | 100 |
|  | Item 10 | 364 | 337 | 93% | 0.0 | 0.0 | 25 | 0 | 40 | 63 | 82 | 83 | 93 | 95 | 91 | 100 | 100 |
|  | Item 2 | 369 | 335 | 91% | 0.0 | 0.0 | 25 | 0 | 33 | 50 | 70 | 77 | 84 | 91 | 98 | 100 | 100 |
|  | Item 1 | 371 | 334 | 90% | 0.0 | 0.0 | 50 | 0 | 67 | 50 | 55 | 65 | 87 | 91 | 96 | 98 | 100 |
|  | Item 7 | 370 | 329 | 89% | 0.0 | 0.0 | 25 | 0 | 40 | 38 | 60 | 81 | 75 | 86 | 98 | 98 | 100 |
|  | Item 11 | 369 | 312 | 85% | 0.0 | 0.0 | 25 | 0 | 40 | 38 | 60 | 84 | 72 | 71 | 87 | 94 | 100 |
|  | Item 12 | 371 | 298 | 80% | 0.0 | 0.0 | 0 | 0 | 50 | 63 | 27 | 48 | 61 | 74 | 87 | 89 | 100 |
|  | Item 6 | 360 | 273 | 76% | 0.0 | 0.0 | 0 | 0 | 0 | 25 | 13 | 28 | 67 | 62 | 75 | 96 | 100 |
|  | Item 8 | 368 | 274 | 74% | 0.0 | 0.0 | 0 | 0 | 0 | 25 | 45 | 40 | 32 | 64 | 84 | 89 | 100 |
|  | Item 5 | 363 | 253 | 70% | 0.0 | 0.0 | 0 | 0 | 20 | 13 | 18 | 23 | 42 | 64 | 63 | 83 | 100 |
| Most Difficult | Item 3 | 367 | 202 | 55% | 0.0 | 0.0 | 0 | 0 | 0 | 0 | 18 | 12 | 16 | 25 | 43 | 65 | 100 |
| Total Score | % |  |  | 100.0 | 0.8 |  | 1.1 |  | 1.6 | 2.1 | 3.0 | 7.0 | 8.6 | 15.6 | 12.3 | 14.8 | 33.2 |
|  | (n, cases) | 373 | 373 |  | 3 | 0 | 4 | 0 | 6 | 8 | 11 | 26 | 32 | 58 | 46 | 55 | 124 |
